# Acute stress responses in Chinese soldiers performing various military tasks

**DOI:** 10.1186/1752-4458-8-45

**Published:** 2014-11-20

**Authors:** Peng Huang, Tengxiao Zhang, Danmin Miao, Xia Zhu

**Affiliations:** Department of Medical Psychology, Fourth Military Medical University, 169 Changle West Road, Xi’an, 710032 China; Institute of Psychology, Chinese Science Academy, 16 Lincui Road, Chaoyang District, Beijing 100101 China

**Keywords:** Chinese soldiers, Major tasks, Acute stress

## Abstract

**Background:**

To examine Chinese soldiers’ acute stress responses, we did this study.

**Methods:**

The soldiers completed the Acute Stress Response Scale (ASRS) when engaged in major tasks, such as earthquake rescue in Wenchuan, Sichuan, and maintaining social stability in Urumchi, Xinjiang. The ASRS has good reliability and validity. The study enrolled 1,832 male soldiers.

**Results:**

The results showed significant differences among five dimensions and the overall response index when comparing four diverse military tasks. Further analysis found that reduced work efficiency and 24 symptom clusters were significantly positively correlated.

**Conclusions:**

The acute stress response of soldiers performing various tasks was influenced by many factors, including the task characteristics and external factors. In addition, the acute stress response affected their work efficiency.

## Introduction

In recent years, natural disasters and terrorist attacks have increased worldwide, and China has not been spared [1, 2]. For example, there was a magnitude 7.0 earthquake in Ya’an in western Sichuan province on April 20, 2013, and a magnitude 8.0 earthquake in Sichuan on May 12, 2008. In addition, there have been many attacks by separatists, extremists, and terrorists. For example, the Kunming Massacre on March 1, 2014, resulted in 33 civilian deaths and more than 140 others were injured ( http://en.wikipedia.org/wiki/2014_Kunming_attack). On April 24, 2013, terrorists armed with axes, knives, and guns burnt down a house in Xinjiang, killing 21 people. When disasters or terrorist attacks happen in China, the People’s Liberation Army (PLA) is the first to respond. More than 13,000 Chinese soldiers have been deployed for rescue and disaster relief assistance since the Ya’an magnitude 7.0 quake ( http://english.people.com.cn/90882/8223005.html).

A national defense white paper on China’s armed forces was released on April 16, 2013; the Diversified Employment of China’s Armed Forces was published by the Information Office of the State Council, the People’s Republic of China (please refer to http://eng.mod.gov.cn/Database/WhitePapers/). In this white paper, the government identified the new situations, challenges, and missions facing the armed forces. In addition, the size of the People’s Liberation Army, Navy and Air Force have been reported publicly. Today, the world faces new challenges regarding peace and development. The white paper stated, "It is a historic mission of these times for the peoples of all nations to grasp the opportunities firmly, meet the challenges jointly, maintain security cooperatively, and achieve development collectively".

As many soldiers are not engaged in war or conflicts, they are required to do other tasks. Such diverse military tasks have become important duties for the PLA [3]. In western countries, especially the United States, the police and firemen are deployed to handle emergencies. By contrast, in China, the PLA has become the main force used to deal with major events such as earthquake rescue.

When soldiers experience acute stress for a long time, it is difficult for them to complete their tasks, either military or non-military. Consequently, in China, we need to study the mental health of soldiers [4, 5], so that they can recover more quickly. Many studies have examined the psychological stress that soldiers face while performing different military tasks [3]. Many researchers have examined related concepts, including acute stress disorder, acute stress reaction, and combat stress reaction [6, 7].

Specifically, acute stress disorder (ASD), generally considered as acute stress reaction, may arise from traumatic experiences and can be developed to post-traumatic stress disorder (PTSD) [6]. The acute stress response, also known as the fight-or-flight response, is occurred under psychologically or physically terrifying circumstances. According to Solomon [8], combat stress reaction is a unique disorder for soldiers with psychic trauma in wars. It significantly impedes many aspects of their daily life. While most studies have focused on PTSD, individuals are also vulnerable to acute stress disorders or acute stress reactions other than PTSD. Robert *et al*. [9, 10] believe that acute stress disorder symptoms affect physical recovery.

Therefore, this study evaluated 1913 PLA soldiers assigned to four different major tasks using the ASRS to reveal the characteristics of their acute stress reactions, to promote faster recovery in the future.

## Methods

### Participants

Inclusion criteria for the soldiers are that, the recruited soldiers must complete at least junior high school education and were deployed for one of the following four different major tasks: earthquake rescue (ER) in Sichuan; plateau training (PT) in Gansu; maintaining social stability (MSS) in Xinjiang; and outside intensive training (OIT) in Henan. 1913 male soldiers, with a mean age of 21.4 ± 3.35 years, were recruited in this study. All the participants have signed the informed consent two days ago before they participated in this study, which was in accordance with the Declaration of Helsinki and all experiments protocol were approved by ethics committee in Fourth Military Medical University, Xi’an, China. Further details are shown in Table 1.

**Table 1:** **The demographic details of the soldiers**

Group	N	Age (M ± SD)	Education degree (n)
Earthquake rescue (ER)	518	22.37 ± 4.82	Junior high school (470), Senior high school (43), no reported (5)
Maintaining social stability (MSS)	210	21.04 ± 3.35	Junior high school (142), Senior high school (56), no reported (14)
Plateau training (PT)	187	20.43 ± 2.36	Junior high school (139), Senior high school (36), no reported (12)
Outside intensive training (OIT)	917	21.76 ± 2.86	Junior high school (762), Senior high school (135), no reported (20)

### Measures

The Acute Stress Response Scale (ASRS) was created by Xia Zhu and her colleagues [11], and was used to measure the soldiers’ mental health. The ASRS has 112 items, with good reliability and validity. There are two main components of the ASRS. The first examines five measurement dimensions (cognitive changes, emotional responses, behavioral changes, physiological changes, and psychiatric symptoms), and the other is the criterion validity dimension (reduced work efficiency). Test-retest reliability of all dimensions is between 0.78 and 0.86. Meanwhile, concurrent validity of all dimensions and the total score for ARSR is significantly positive relationship with the total score for SCL-90. Of the subjects, 1905 completed the ASRS and of these 1832 were complete and could be analyzed in this study.

### Procedure

The ASRS was distributed to the soldiers after they had been performing their tasks for 5–10 days, a period sufficient to lead to an acute stress response. The data were analyzed using SPSS ver. 16.0, including general descriptive statistics and repeated measures analysis of variance (ANOVA).

## Results

### Comparison of the four major tasks

**Figure 1:**
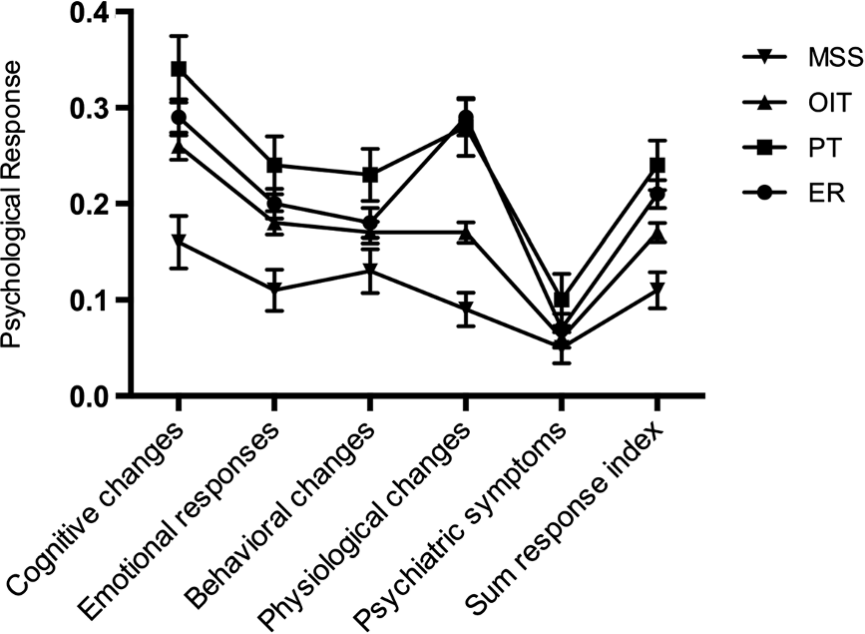
**The change trends of psychological responses according to different groups.** Error bar (Mean with 95% confidence interval (CI)) has been added for each data point.

Table 2 and Figure 1 summarize the acute stress responses to the four major tasks.

**Table 2:** **The comparison of each psychological response according to different groups**

Group	N	Cognitive changes	Emotional responses	Behavioral changes	Physiological changes	Psychiatric symptoms	Sum response index
ER	518	0.29 ± 0.22	0.20 ± 0.18	0.18 ± 0.18	0.29 ± 0.22	0.07 ± 0.18	0.21 ± 0.17
PT	187	0.34 ± 0.24	0.24 ± 0.21	0.23 ± 0.19	0.28 ± 0.21	0.10 ± 0.19	0.24 ± 0.18
MSS	210	0.16 ± 0.20	0.11 ± 0.16	0.13 ± 0.17	0.09 ± 0.13	0.05 ± 0.12	0.11 ± 0.14
OIT	917	0.26 ± 0.22	0.18 ± 0.19	0.17 ± 0.17	0.17 ± 0.16	0.06 ± 0.15	0.17 ± 0.15
F		24.42***	17.52***	11.80***	82.79***	4.03***	28.96***

***p < 0.001.

There were significant differences among the five dimensions and the overall response index. The cognitive changes were the most obvious, followed by the emotional responses.

Comparing the four tasks using ANOVA, there were significant differences in the overall response index (F = 28.96, *p* < 0.001, *ƞ*^2^ = 0.05). In the multiple comparisons, PT and ER had higher scores, and OIT and MSS lower ones, but there were no significant differences among the four tasks. In addition to the overall response index as the dependent variable, ANOVA was performed with the five dimensions as dependent variables. This showed significant differences in the cognitive changes (F = 24.42, *p* < 0.001, *η*^2^ = 0.04), emotional responses (F = 17.52, *p* < 0.001, *η*^2^ = 0.03), behavioral changes (F = 11.80, *p* < 0.001, *η*^2^ = 0.02), and psychiatric symptoms (F = 4.03, *p* < 0.01, *η*^2^ = 0.007). The scores were in the order PT > ER and OIT > MSS. For physiological responses (F = 82.79, *p* < 0.001, *η*^2^ = 0.12), multiple-comparison showed that the scores were in the order PT and ER > OIT > MSS.

### Criterion validity

With reduced work efficiency as the criterion validity, the details of the four groups are summarized in Table 3. There were significant differences among the four tasks (F = 22.52, *p* < 0.001, *η*^2^ = 0.04, ANOVA). In the multiple comparison, ER and PT > OIT and MSS.

**Table 3:** **Reduced work efficiency in different groups**

Group	N	M	SD
ER	518	0.33	0.42
PT	187	0.37	0.43
OIT	917	0.25	0.36
MSS	210	0.11	0.26

Further analysis showed that reduced work efficiency and 24 symptom clusters were significantly positively correlated (r = 0.14 ~ 0.58, *p* < 0.001). With reduced work efficiency as the dependent variable and the 24 symptom clusters as independent variables, stepwise regression (n = 1832) was conducted. The resulting equation included seven symptom clusters (anxiety, somatic symptoms, attention loss, guilt, apathy, psychiatric symptoms, and anger), which explained 45% of the population variance (Table 4).

**Table 4:** **Stepwise regression analysis of 7 symptom clusters impact on work efficiency**

Symptom clusters	β	p	△***R*** ^2^
Anxiety	0.25	0.000	0.345
Somatic symptoms	0.24	0.000	0.063
Attention loss	0.15	0.000	0.022
Guilt	0.12	0.000	0.01
Apathy	0.06	0.008	0.002
Psychiatric symptoms	-0.05	0.019	0.002
Anger	0.05	0.049	0.001

### Psychological response of different tasks

Table 5 shows the results combining the four major tasks (n =1832). As Table 5 shows, the overall response index is 0.18 ± 0.16, and cognitive changes had the highest score (0.27 ± 0.22) and psychiatric symptoms the lowest (0.07 ± 0.16).

**Table 5:** **The psychological response result of four major tasks**

Psychological response	M	SD
Cognitive changes	0.27	0.22
Emotional responses	0.19	0.19
Behavioral changes	0.18	0.18
Physiological changes	0.21	0.19
Psychiatric symptoms	0.07	0.16
Sum response index	0.18	0.16

## Discussion

As the PLA soldiers perform stressful tasks frequently, they may experience ASR more often. Results show that different tasks may lead to different degree of acute stress reaction. Psychological stress has been studied in detail since the late twentieth century. Dabhar and McEwan [12] found that chronic stress is immunosuppressive, while acute stress is immune enhancing.

In the plateau training task, the soldiers had the highest overall response index scores, which meant that the soldiers experienced the most severe acute stress among the four tasks. The most likely causes are the tough training, combined with the physical discomfort and lack of oxygen. Most tended to undergo cognitive changes, followed by emotional responses. Most of the soldiers suffered negative emotions, such as anxiety, guilt, apathy, and anger. Therefore, for the soldiers, cognition and emotion regulation are important.

In addition, their acute stress reactions will influence the soldiers’ work efficiency, which is a key to completing the four tasks. We found that reduced work efficiency and 24 symptom clusters were significantly positively correlated; the negative emotions included anxiety, somatic symptoms, attention loss, guilt, apathy, psychiatric symptoms, and anger. Our results were similar to those of Hu *et al*. [3], who used questionnaires to study the psycho-physiological response of Chinese soldiers performing diverse tasks. The ASRS is a simple, effective tool that can accurately assess the psycho-physiological stress response [11].

The utilization of ASRS has three main implications. Firstly, the ASRS is an effective tool to monitor the soldiers’ stress level when they are assigned to major tasks. For the command department, the leaders can directly and timely obtain the soldiers’ stress responses status so that they are able to make better decisions, for example, withdraw the high stress soldiers to the rear.

Secondly, the use of ARSR is not limited to soldiers, but other groups of people as well, for instance, rescue personnel, survivors and even workers. People can evaluate their acute stress response by using ARSR in order to examine their psychological states before things getting worse.

Thirdly, as the number of reported natural and manmade disasters increases dramatically [13], people who have related experience are more likely to have psychological disorders, like ASR, PTSD. Our scale is designed to estimate the relationship between ASR and resilience and relationship between ASR and PTSD and it can be a complement physiological and biomedical indicator [11].

However, this study also has some limitations. Firstly, we only studied four distinct groups and we will include more category groups in future study. Secondly, the data collection of this study is not efficient enough and we will develop a special mobile application to enhance the efficiency.

## Conclusions

In summary, soldiers who participate in major tasks will experience a variety of acute stress reactions. The degree of the acute stress response will differ according to the type of task, but the main changes are reflected in the cognitive and emotional reactions. Therefore, we should pay more attention to the characteristics of acute stress reactions and adopt targeted interventions rather than allow the acute stress reaction to progress to post-traumatic stress disorder [14, 15]. It is important to eliminate the effect of stress to improve the soldiers’ performance of diverse military tasks. Data on acute stress response characteristics should be collected to monitor changes in soldiers’ reactions and to devise different targeted interventions for mild, moderate, and severe stress responses.
